# Longitudinal Protective Factors against Intimate Partner Violence for Women Born in Australia and Women from Refugee Backgrounds

**DOI:** 10.3390/women4030024

**Published:** 2024-09-09

**Authors:** Ruth Wells, Louis Klein, Mohammed Mohsin, M. Claire Greene, Jane Fisher, Derrick Silove, Zachary Steel, Susan Rees

**Affiliations:** 1 Discipline of Psychiatry and Mental Health, School of Clinical Medicine, Medicine and Health, University of New South Wales, Sydney 2052, Australia;; 2 Program on Forced Migration and Health, Heilbrunn Department of Population and Family Health, Mailman School of Public Health, Columbia University, New York, NY 10027, USA; 3 School of Public Health and Preventive Medicine, Faculty of Medicine, Nursing and Health Sciences, Monash University, Melbourne 3800, Australia;

**Keywords:** intimate partner violence, protective factors, people from refugee backgrounds

## Abstract

Intimate Partner Violence (IPV) is a risk factor for depressive disorders and other harms to women and their pregnancy. There is a need for longitudinal evidence to assist with understanding the subgroups of women including those from refugee background affected by IPV. We recruited women at their prenatal visit from three antenatal clinics in Australia (January 2015–March 2016). A total of 1335 women, 650 (48.7%) born in Australia and 685 (51.3%) from refugee backgrounds, completed baseline assessment; then, Time 2 follow-up was at 6 months and Time 3 follow-up was at 24 months post birth. The WHO Intimate Partner Violence (IPV) measure was used. Latent class growth analysis grouped individuals based on trajectories of IPV across three time points. A three-step process identified characteristics associated with respective latent class membership. Similar three-class solutions were observed across both cohorts, composed of Limited IPV (64% and 48% Australian-born and refugee background, respectively); Changing IPV (31%; 46%)—various combinations of IPV categories across time; Combined IPV (4%; 6%)—IPV at all time points, all transitioning to the combined physical and psychological abuse category at Time 3. Older age, fewer children, being in a couple, having a better partner, family and friend relationships, fewer partner trauma events, and fewer living difficulties emerged as protective factors for the changing and combined categories, with a distinct pattern for the refugee cohort. The findings assist with understanding and defining of the highest risk group for targeting interventions to prevent IPV, and the unique protective factors across the two IPV-affected classes for women born in Australia and those who arrived as refugees.

## 1. Introduction

Intimate Partner Violence (IPV) is associated with an elevated risk for major depressive disorder, suicidality, and functional impairment [1]. There have been calls to better characterise the typologies related to how IPV manifests for women over time, and the interrelated individual, social, and structural factors that may applied to understand and address the risks of IPV [2]. Unfortunately, the predominantly cross-sectional research has tended to focus on the individual characteristics of at-risk women experiencing IPV, rather than the how patterns of IPV may change or manifest over time and the related socio-cultural and protective factors [3]. In fact there is limited longitudinal evidence focusing on the contextual factors that are protective against IPV, including those related to the male partner as well as social or structural factors [3,4].

The current research aims to identify trajectories of IPV experiences over three years, including protective factors associated with a reduced risk of IPV among 1335 women (650 Australian-born and 685 women from refugee backgrounds) recruited through antenatal clinics in Australia. Antenatal services are a key site where many women encounter the health system and are therefore uniquely placed to prevent IPV. In addition, IPV during the perinatal period is associated with maternal perinatal depression and post-traumatic stress symptoms [5].

We take an intersectional feminist perspective, which recognises the unique psychological and social experiences [6,7] of women from refugee backgrounds [8]. Conflict-affected populations (including people from refugee backgrounds), especially women, have been shown to report higher levels of post-traumatic stress disorder and depression related to potentially traumatic events (PTEs), as well as higher rates of IPV associated with conflict [9,10]. Of note is the impact of traumatic events on the experiences of, and perpetration of, IPV [11,12] especially among migrants [13,14].

A recent review on exposure to gender-based violence among adolescent girls in humanitarian settings [15] has shown how factors across the ecological layers increase the risk of experiencing violence. This includes (1) Individual: witnessing violence and increased mental health problems; (2) Relational: separation and loss of loved ones and changes to family structure; (3) Community: reduced social supports and community-wide exposure to violence; and (4) Structural: increasingly rigid gender roles and weakened education systems. In resettlement countries, postmigration living difficulties, such as not speaking the language, discrimination, and separation from family, continue to play a role in impairing psychosocial functioning in high-income countries [16,17].

We used latent class growth analysis as a robust person-centred method for identifying groups of individuals who experience different patterns of IPV over time [18]. This data-driven approach allocates people to groups with similar IPV trajectories over time. We then used a hypothesis-driven ecological approach to determine the sociodemographic characteristics that are associated with the latent trajectory classes of IPV.

We hypothesised that women who experienced IPV across three time points are more likely to be younger [3,19–21], have experienced more trauma in their lives (PTEs) [22], have more children [23] have fewer supportive family and friend relationships, partners who have experienced more traumatic events (PTEs) [24], and more financial stress [25]. We also assumed that the women from refugee backgrounds who reported IPV across three timepoints would have higher levels of post-migration living difficulties [26]. We considered separate models for Australian-born women and women from conflict-affected countries.

## 2. Results

### 2.1. Participants

At baseline (T1), 1335 of the 1574 eligible women (84.8% response rate) were interviewed, including 650 (48.7%) women born in Australia and 685 (51.3%) from refugee backgrounds. At Time 2 (T2), a total of 1111 (retention rate 83.2%;) were re-interviewed, including 528 (47.2%) women born in Australia and 583 (52.5%) from refugee backgrounds. At Time 3 (T3), 930 (retention rate 69.7%) were re-interviewed, of whom 447 (48.1%) were Australian-born and 483 (51.9%) were born in conflict-affected countries. Overall, 905 women (overall retention rate 67.8%) were interviewed at all three time points, including 435 (48.1%) Australian-born and 470 (51.9%) from refugee backgrounds (see also Supplementary Table S1).

### 2.2. Sociodemographic Characteristics

The mean age at T1 was 29 years (SD 5.4) for Australian-born women and 30 years (SD 5.4) for women from refugee backgrounds (See Table 1 sample characteristics within each IPV group). The following sociodemographic characteristics were reported by Australian-born women and women from refugee backgrounds, respectively: 66% and 70% had previous children; 76% and 81% were in a couple relationship; 49% and 77% reported potentially traumatic events; 88% and 86% could rely on their partner a lot or some; 81% and 50% could rely on three or more family members; 53% and 29% could rely on three or more friends; 77% and 62% reported no financial stressors; and the mean number of postmigration living difficulties reported by refugee-background migrant women was 1.9 (SD 2.5).

### 2.3. Latent Class Growth Analysis

The goodness-of-fit indices for models with 1 to 6 classes for refugee-background and Australian-born samples are shown in Table 2 for imputed data and Supplementary Table S3 for observed data. Both the refugee-background and Australian-born cohorts returned a three-class solution (Limited IPV, Changing IPV, and Combined IPV) for observed data and imputed data. The testing of a growth mixture model resulted in convergence problems, so LCGA (in which within-class intercepts and slopes are constrained) was used (Table 3). Given the sufficient sample size [27] and lack of convergence issues with the LCGA model, we did not fix the variance–covariance between classes. Linear and quadratic trajectories were tested with 20 random starts. Analyses were run with 150 imputed datasets and the best log-likelihood was replicated.

### 2.4. Composition of Latent Classes

Two robust latent class solutions from both imputed and observed data were identified from across Australian-born and refugee-background samples. The three classes for Australian-born and refugee-background, respectively, as follows: (1) Limited IPV (Observed *n* = 423, 65%; *n* = 356, 52%; Imputed *n* = 417, 64%; *n* = 330, 48%) at most time points; (2) Changing IPV (Observed *n* = 208, 32%; *n* = 302, 44%; Imputed *n* = 204, 31%; *n* = 315, 46%)—various combinations of IPV categories across time; and (3) Combined IPV (Observed: *n* = 19, 3%; *n* = 27, 4%; Imputed *n* = 29, 4%; *n* = 41, 6%)—IPV at all three time points, all those transitioning to the combined physical and psychological abuse category at time 3. The frequencies, intercepts, and slopes for each IPV latent class are presented in Table 1 for imputed and observed data.

**Figure 1.**
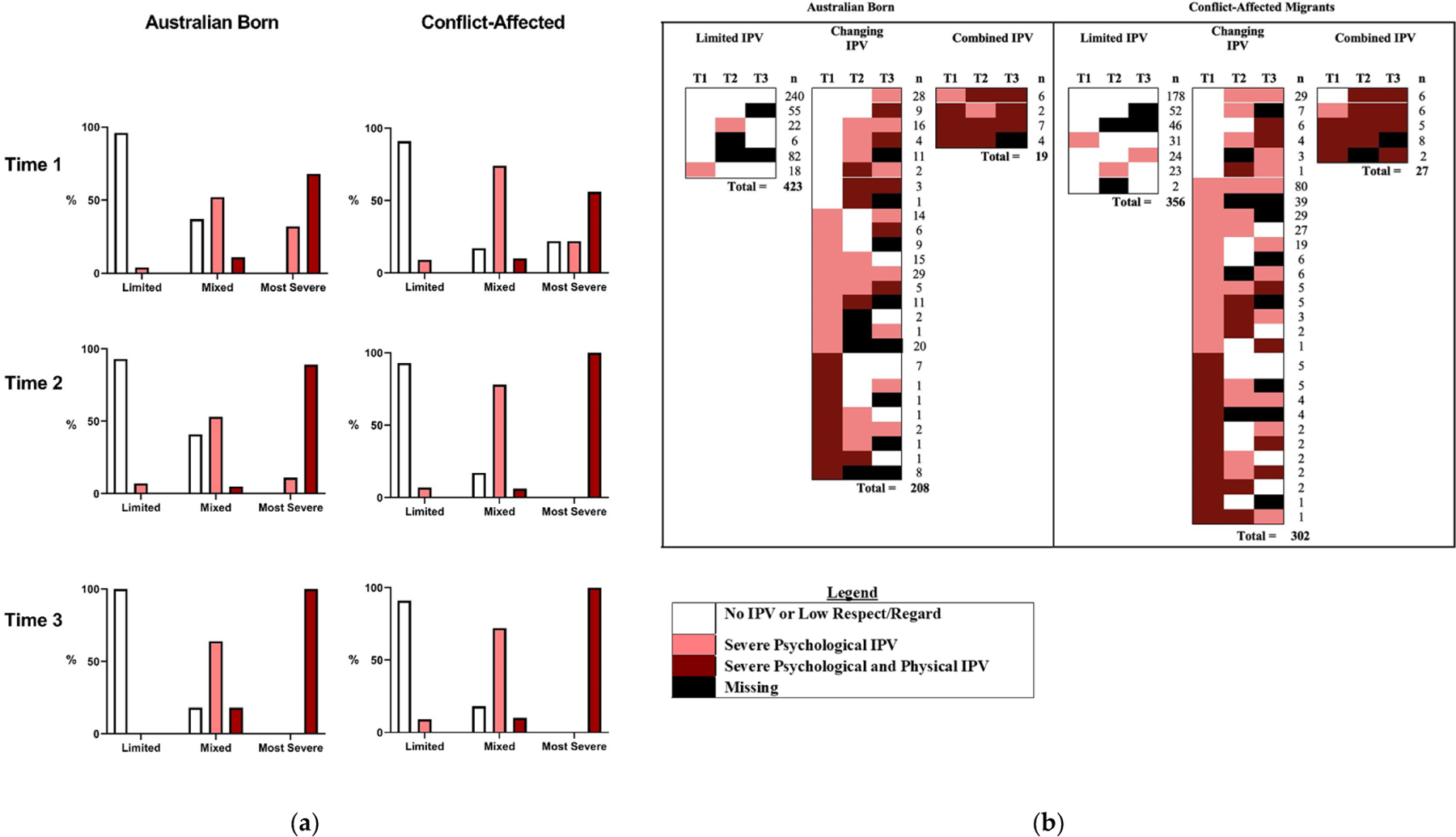
Patterns of IPV reporting over time. Note. White columns indicate no IPV reported or low respect/regard only; pink indicates severe psychological IPV; and red indicates combined severe psychological and physical IPV. (**a**) Percentage of women in most likely latent classes reporting IPV types at each time point from observed data. Presented as a percentage of people in each latent class reporting different forms of IPV at each time point. (**b**) Shows all patterns of reporting, including missing data within each latent class for observed data. Numbers to the right indicate the number of people reporting the depicted pattern for each combination. The total *n* for the class is listed below each class. People in the Limited IPV group report either no IPV or low respect/regard at all time points, or either missing or psychological IPV at other time points. People in the Changing group reported a wide variety of trajectories, with most people moving between categories of IPV. People in the IPV class all reported combined psychological and physical IPV at a minimum of two timepoints and all transitioned to combined IPV or missing at the final time point.

For the observed data, the posterior probabilities were used to assign each individual to their most likely latent class. The distribution of IPV categories at each time point and their relation to sociodemographic characteristics are presented in Table 1. The percentage of each IPV type at each time point and the patterns of IPV reporting and missing data at Time 2 and Time 3 are depicted in Figure 1 (see also Supplementary Table S3).

#### Step Process Multinomial Logistic Regression Using Imputed Data

Table 4 reports the adjusted odds ratios (AORs) from multinomial logistic regression analysis for the sociodemographic variables associated with IPV latent class membership. The results for the imputed data are presented, as they are based on the three-step process that takes into account classification errors in the initial step.

### 2.5. Refugee-Background Sample

#### Changing IPV compared to Limited IPV.

The following variables were protective, that is, protected women from being in the *Changing* group compared to being in the Limited group: being older; having two or fewer children; being able to rely or confide in a partner some or a lot; having three or more friends to rely or confide in; having fewer partner PTEs; and reporting no financial stressors.

#### Combined IPV compared to Limited IPV.

Factors that were protective for women against being in the most severe category (Combined IPV) compared to Limited IPV were being of an older age, having two or fewer children, being able to rely or confide in a partner some to a lot, reporting fewer partner PTEs, and with lower number of living difficulties

#### Combined IPV compared to Changing IPV.

Factors more likely to be protective against being in the Combined IPV group compared with IPV that changed in type or reported occurrence overtime (Changing IPV) were living in a couple family, able to rely or confide in a partner, and having lower number of living difficulties.

### 2.6. Australian-Born

#### Changing IPV compared to Limited IPV.

Factors that were protective for women against being in the Changing IPV category compared to Limited IPV were being an older age, not having experienced PTEs, having three or more friends to rely or confide in, and being able to rely or confide in a partner.

#### Combined IPV compared to Limited IPV.

Factors more likely to be protective against being in the most severe Combined IPV group compared with IPV in the Limited IPV category were having three or more friends to rely or confide in, being able to rely or confide in a partner, and having no financial stress were protective against combined IPV.

#### Combined IPV compared to Changing IPV.

Having three or more friends to rely or confide in and being able to rely or confide in partner were protective against being in the Combined IPV category compared with the Changing IPV category.

## 3. Methods

### 3.1. Participants and Recruitment

The participants for the WATCH (Women Aware Together with their Children) longitudinal cohort study were recruited between January 2015 and March 2016. We aimed to recruit women from conflict-affected countries representing the highest intakes from the Middle East, South Asia, and African regions. Regardless of the visa for entry, all Arabic-speaking women from conflict-affected countries were approached, with the final sample being predominantly from Iraq, Lebanon, Sri Lanka, and Sudan. A randomly selected comparison group of women born in Australia were also recruited. We recruited from three public antenatal clinics during the women’s first appointment (generally 12–20 weeks’ gestation) in the cities of Sydney, New South Wales, and Melbourne in Victoria, Australia. Three study sites were selected due to their positioning within areas known to have substantial populations of refugees from conflict-affected regions and that were otherwise largely representative of the general population. For more details on the recruitment procedure, please see [9]. Women with overt psychosis, severe medical illness, and obvious intellectual impairment were excluded. Women members of the research team who spoke the same language as eligible women approached them in the waiting room and conducted interviews (up to 1 h) in private areas with the consenting women. Research assistants were trained to be consistent with the WHO guidelines to ensure women were in a separate and private room or space during the face-to-face or telephone interview. The same strategy was used to ensure that women were in private during subsequent phone interviews. If privacy could not be obtained, the IPV questions were omitted. The *N*s of men who refused to leave their partner or did not support them to participate were recorded as a refusal (possible partner coercion). Concerns for women in relation to possible domestic violence were shared with the hospital social worker. At baseline (Time 1), 650 Australian-born and 685 women from refugee backgrounds were interviewed (the response rate was 84.8%; 1335 out of 1574). Approximately 6 months and 24 months after the birth of the index child, two consecutive follow-up surveys (Time 2 and Time 3) were conducted at home either by telephone or in person (where telephone interviews were not possible). The most common reason for non-participation in follow-up surveys were being uninterested in the study, followed by being too busy, feeling unwell, and hostility from partners or relatives.

### 3.2. Ethics and Research Personnel

The study was approved by the Human Research Ethics Committee, Liverpool Hospital, Australia, the Southwestern Sydney Local Health District Human Research Ethics Committee (HREC/15/POOL/28), and the Monash Health Ethics Committees. Participants provided written informed consent and were remunerated for their time. Eight bilingual women fieldworkers were given extensive training [28]. The World Health Organization (2007) guidelines for conducting safe and ethical IPV research and the Strengthening the Reporting of Observational Studies in Epidemiology (STROBE) reporting guidelines [29] were followed.

### 3.3. Survey Measures

All measures were selected based on previous psychometric evaluations across cultures. After standard translation and back-translation procedures were performed, final refinements were made by groups of linguistic experts.

#### 3.3.1. Sociodemographic Characteristics

The Australian National Census items were adopted to assess sociodemographic characteristics. Data collected at Time 1 included age in years; financial stressors (0 = none, 1 = 1 or more); family composition (couple = 0; single parent or multiple family = 1); number of children before current pregnancy (2 or fewer; 3 or more); migrant or Australian-born; whether the participant can rely or confide in their spouse (0 = a lot/some, 1 = little/not at all); and how many friends/family members they can rely or confide in (0 = three or more, 1 = two or fewer).

#### 3.3.2. Potentially Traumatic Events (PTEs)

The lifetime exposure to 18 PTEs, based on the World Mental Health Survey [30], was collected for women at Time 1. For women who reported PTEs for partners, data were also collected at Time 2. See Supplementary Table S2 for PTE items. Items were coded 1 for yes, and 0 for no for lifetime exposure. Based on the distributions, we grouped scores into two groups for women (none vs. 1 or more events) and four categories for men (none; one; two to three; and four and more).

#### 3.3.3. Intimate Partner Violence

We applied the World Health Organization (WHO) measures for IPV that have been used across 14 countries globally, and enquire into lifetime physical, psychological, and sexual violence perpetrated by a current or past intimate partner [31]. Cultural experts advised against including explicit sexual abuse items because religious and traditional values can make discussing issues related to sex disrespectful, shameful, or traumatising. We aimed to interview women about sensitive issues in a respectful and supportive way, without alienating them. We do include an item which relates to having something done to you that you do not or cannot speak about. We assigned women to one of the following three categories: (1) No IPV or low respect or regard items only; (2) psychological IPV (without physical abuse, including jealous or angry behaviour if she talks to other men, frequent accusations of being unfaithful, does not permit meetings with female friends, limits contact with family, insists on knowing woman’s whereabouts, humiliates her in front of others, and/or threatens harm to her or someone close to her); and (3) psychological IPV and physical IPV (any physical abuse including pushing; shaking; throwing items; slapping; twisting arm; punching; kicking; dragging; strangling; burning; threats with a knife, gun, or other weapon; and attacks with a knife, gun, or other weapon).

#### 3.3.4. Post Migration Living Difficulties

The Post Migration Living Difficulties Checklist (PMLD) measures migration stressors [26] and was administered to refugee-background women only. The 21-item list used in the current study was adapted from items based on research with migrants and refugees in Australia [32] and included items related to communication; discrimination; family separation; worry for family back home; not being able to return home; employment; detention; reduced access to health and social services; isolation; and fear of repatriation. Additional items advised by cultural experts and related to IPV were included: worry about being sent home by your partner; fear partner will take your children; partner might marry or live with another woman; and problems related to dowry. Participants answered on a 3-point scale, as follows: (0): no problem at all; (1): a problem; and (2): a very serious problem. The total number of items rated as a very serious problem were summed to generate a total score. Cronbach’s alpha (α) for the item pool of PMLD was 0.80 at the first follow-up and 0.77 at the second follow-up survey. See Supplementary Materials for a full list of items.

### 3.4. Statistical Analysis

Latent class growth analysis (LCGA) groups individual trajectories of change in IPV over time into latent classes based on their patterns of change in the data over time [33]. LCGA is a special case of longitudinal mixture modelling, in which within-class variability is constrained to zero. A complete case analysis was not possible due to missing data (see Supplementary Table S1). Missing T2 and T3 was assumed to be missing-at-random (MAR) and was imputed using multiple imputation by chained equations (MICE) [34] using the R language v3.6.1 [35] within RStudio IDE 4.2.1 [36]. MICE was used so that missing data could be imputed with reference to demographic characteristics that could influence why they were missing. This is more robust than simply using the full information matrix. See Supplementary Materials for details of the methods and for the full specification and code.

This paper follows the GRoLTS checklist for reporting on latent trajectory studies [37]. LCGA was conducted at times 1, 2, and 3 in Mplus 8 [38] with the three-group ordered categorical outcome variable of (1): No IPV or low respect/regard; (2) psychological abuse; and (3) psychological and physical abuse. Trajectories across the time points were used to assign participants to a latent class based on posterior probabilities. Iterations of the model were performed with increasing numbers of latent classes and their model fit was compared to identify the best fitting model. Linear and quadratic models were tested [37]. The best model is selected based on both theoretical considerations and relative statistical fit, according to Bayesian Information Criterion (BIC), to compare log-likelihood [27], whereby a difference of 10 indicates that an additional class makes a meaningful contribution [39], and sample-size-adjusted BIC (SS-BIC) [40]. Entropy measures the probability that participants are correctly classified into classes, with values closer to 1 indicating a better accuracy, with 0.7 indicating an acceptable classification. The most parsimonious model was selected.

#### Association with Sociodemographic Variables

We used a three-step procedure to examine the association between latent classes and sociodemographic auxiliary variables. Auxiliary variables are variables hypothesised to be associated with the latent classes, but are not used to determine class membership. The three-step process is recommended for auxiliary variables, as adding them as covariates to the model changes the classification process [41]. The three steps include (1) running the latent class model; (2) assigning individuals to latent classes based on posterior probabilities; and (3) regressing the most likely latent class on auxiliary variables, taking into account misclassification in the second step [33]. Auxiliary variables represented a range of ecological domains including individual (age and PTEs); relational (number of children, family composition, spouse, friend and family social support, and partner PTEs); and structural (financial stress and postmigration living difficulties [refugees only]).

## 4. Discussion

We identified three distinct trajectories of IPV reported by women across the 3-year period both across the refugee-background and Australian-born cohorts. These included Limited IPV (48 and 64%, respectively); Changing IPV (46 and 31%); and Combined (6 and 4%) IPV. We simultaneously examined protective factors for each category, taking an ecological approach that included individual, relational (relationship and interpersonal), and structural levels. This novel approach fills an important gap in the literature by examining the factors that are associated with IPV trajectories after taking ecological factors into account. Relational factors, in particular concerning the male partner, and structural factors (e.g., migration stressors and financial stress) emerged as key factors influencing trajectories. Health practitioners routinely screen pregnant women for IPV. These findings highlight that a preventative approach must take a comprehensive range of individual, interpersonal and structural factors into account.

Consistent with multi-country cross-sectional research [42], we identified a small group of women (Combined group) who experienced both psychological and physical IPV, which persisted across the three timepoints. The Combined category of abuse indicates those most at risk and is associated with worse health outcomes for women, higher numbers of controlling acts, and more physical abuse [43]. This finding underscores the importance of the targeted, early identification of these women at an arguably greatest risk of life endangerment [44,45].

The Changing group represents a large, heterogenous group of women in this study who report changing patterns of IPV type and/or occurrence across time. This finding shows that measuring IPV at a single time point may fail to capture the magnitude of the population of at-risk women, or the nature of the women’s experiences as they and their families undergo different life stresses.

### 4.1. Protective Factors

An older age during pregnancy was protective, a finding which may also be associated with a reduced likelihood to disclose past IPV with age [46]. Research in some of the conflict-affected countries included in this study has found that a younger age of marriage is associated with a risk for IPV, having less education, and worse mental health outcomes [21,47]. Women’s PTEs (fewer PTEs) was protective only in the Australian-born cohort when comparing the Changing and Limited IPV groups. While past trauma has been associated with a risk of IPV in multiple cross-sectional studies [24], it is interesting to note that women’s past trauma did not emerge as significant when taking the full range of factors into account. For women from refugee backgrounds, this effect may be related to the small numbers of women in all groups who had not experienced PTEs. For both cohorts, this may be because the experience of IPV was more closely related to other factors (such as current financial stress or post-migration living difficulties).

For refugee-background women, family composition factors (being in a couple and having less children) were protective from being in the IPV groups, but this was not the case for Australian-born women. Of note is that women who did not consider themselves to be in a couple relationship at the start of the study may have been at risk of IPV from previous partners. It may be that Australian-born women generally have more access to reliable family (81%) and friend (53%) support than migrant women (50% family and 29% friends), and so have greater resources to help face the challenges of raising multiple children or relationship break-ups [48]. In both the refugee-background and Australian-born cohorts, moderate or more friend relationships were protective against being in the Changing IPV group. This demonstrates the importance of connecting women with their social networks when supporting them, including to leave abusive relationships [49]. In contrast, friend relationships were not a protective factor from being in the most severe Combined group, where partner and structural factors were more strongly associated. One possible explanation is that access to social support from friends might also be affected by the abusive partner. This is consistent with the literature on coercive control, wherein abusive partners restrict women’s access to social support including friends and family [50]. We further note that fewer friend relationships for the woman may also reflect isolation of the male partner from social and community supports, a factor that has been associated with IPV perpetration [51].

The most consistent protective factor concerned the woman’s partner. A better-rated partner relationship and a lower number of reported partner traumatic events (PTEs) were protective against the IPV groups in nine of twelve comparisons. Practitioners should focus on the characteristics of the male partner in identifying women at risk of IPV and note that signs of a lack of trust in the partner should be further explored with specific and direct questions about whether IPV occurs in the home. We note that partner PTEs were reported by the women, so may not accurately represent the number of PTEs the partner experienced. However, it clearly represents an important phenomenon, which is related to the woman’s interpretation of the partner’s trauma experiences and their relationship with his use of violence. It is important not to make excuses for IPV, as this may further harm the woman involved [52]. However, systematic evidence has identified male partner exposure to political violence as a risk for IPV [21,53]. A trauma-informed approach is needed to appropriately understand and effectively respond to the perpetration of IPV [54], taking into account the stressors that both partners are exposed to [51].

The absence of financial stress was protective for both IPV affected groups for Australian-born women, but not for women from refugee backgrounds. Experiencing financial stress and economic factors may indicate risk for IPV [55,56], but this may not have been significant for women from refugee backgrounds because other post migration living difficulties (which included unemployment) were more significant in their lives. These post-migration living difficulties included discrimination, poor access to services, unemployment, little government help with welfare, poverty, worries about family back at home country and family separation—factors which are likely to affect both the women and their partners—and have been associated with poor mental health outcomes [57]. Programmes which can address economic and social marginalisation and family stress, and which address structural barriers to social integration for refugees [4], may help to both address the social determinants of distress and prevent Combined IPV. The need for this is especially acute in the current context of the pandemic and ecological crisis in Australia [58].

### 4.2. Limitations

Methodologically rigorous techniques for the multiple imputation of missing data, subsequent analysis applying LCGM, and the inclusion of a salienta range of protective factors are key strengths. Several limitations should nonetheless be noted. Changes in reported IPV between baseline and follow-up interviews could be due to a new onset of IPV since the baseline interview, changes in willingness to report, or changes in recall. Attrition of IPV reporting over time in the Changing category may have been impacted by having a new and non-violent partner or a range of influences including memory and disposition to recall events. We used an imputation approach to ensure that all available data on these women were included and to minimise bias.

Secondly, protective factors were only measured at time point 1, except for partner PTEs, which were reported by the woman at Time 2; as such, time-varying protective factors may have shifted over the course of the data collection period. The women’s reporting of partner PTEs may not have been accurate. Thirdly, despite stabilised estimates, multiple imputation was performed for some variables, with greater than 25% missing data (see Supplementary Material for details). Fourthly, while the Combined IPV group was within recommendations for size, it was nonetheless smaller when compared against the Limited and Changing IPV groups; this is an imbalance that may have influenced which protective factors emerged from the latent class models.

### 4.3. Future Research Directions

Our findings demonstrate the importance of considering the longitudinal course of IPV to understand how IPV can manifest over time and to identify those at the highest risk of experiencing IPV that changes in nature and occurrence overtime, as well as chronic, combined abuse. Future research should build on the latent class growth approach by identifying how IPV changes across the life course and what factors are related to this change. In addition, studies which can link individual and relational data in a multi-level model, including neighbourhood characteristics, will strengthen our understanding of the community-level factors that are associated with IPV.

### 4.4. Prevention, Clinical, and Policy Implications

This study shows that a large group of women may experience IPV that varies in nature over time. This important finding suggests that the right intervention, informed by studies such as this, could logically change a trajectory to prevent future recurrence. This study also shows that there is a smaller group at an elevated risk of experiencing persistent IPV that culminates in exposure to both psychological and physical abuse. This high-risk group needs to be identified and directly targeted for specific interventions. It is essential that women presenting to antenatal clinics are screened for IPV using safe and culturally relevant and sensitive techniques. While the screening and identification of IPV is essential, responses need to be integrated across services (social, medical, and legal) to ensure the integrated support required to address this major public health issue [59]. Training for frontline staff and those developing interventions should include understanding of the protective factors specific to Australian born and refugee background populations, and to target the unique relational (e.g., social supports, interpersonal) and structural (e.g., migration and financial stress) factors that could influence patterns of IPV and mental health in both groups of women.

## Supplementary Material

Supplementary Material

The following supporting information can be downloaded at: https://www.mdpi.com/article/10.3390/women4030024/s1, Table S1: Number of participants interviewed and missed out with retention rate for Australian-born women and women from refugee backgrounds across three time points; Table S2: List of potential traumatic events (PTEs); Table S3: Observed data goodness-of-fit indices for latent growth classes for women from refugee backgrounds and Australian-born women.; code for analyses. Reference [60] is cited in Supplementary Materials.

## Figures and Tables

**Table 1.** IPV and sociodemographic characteristics in relation to latent classes for Australian-born and refugee-background women (most likely latent class membership, observed data only).

			Australian-Born		Combined	Conflict-Affected Countries (*n* = 685)			
		Total	Limited	Changing		Total	Limited	Changing	Combined
			IPV	IPV	IPV		IPV	IPV	IPV
		*n* (%)	*n* (%)	*n* (%)	*n* (%)	*n* (%)	*n* (%)	*n* (%)	*n* (%)
Time 1 IPV	Time 1 Total	650 (100)	423 (100)	208 (100)	19 (100)	685 (100)	356(100)	302 (100)	27 (100)
	No IPV	482 (74.2)	405 (96)	77 (37)	0 (0)	381 (55.6)	325 (91)	50 (17)	6 (22)
	Psychological IPV	133 (20.5)	18 (4)	109 (52)	6 (32)	259 (37.8)	31 (9)	222 (74)	6 (22)
	Physical and Psychological IPV	35 (5.4)	0 (0)	22 (11)	13 (68)	45 (6.6)	0 (0)	30 (10)	15 (56)
Time 2 IPV	Time 2 Total	528 (100)	335 (100)	174 (100)	19 (100)	583 (100)	308 (100)	250 (100)	25 (100)
	No IPV	385 (72.9)	313 (93)	72 (41)	0 (0)	327 (56.1)	285 (93)	42 (17)	0 (0)
	Psychological IPV	117 (22.2)	22 (7)	93 (53)	2 (11)	217 (37.7)	23 (7)	194 (78)	0 (0)
	Physical and Psychological IPV	26 (4.9)	0 (0)	9 (5)	17 (89)	39 (6.7)	0 (0)	14 (6)	25 (100)
Time 3 IPV	Time 3 Total	447 (100)	286 (100)	146 (100)	15 (100)	483 (100)	258 (100)	206 (100)	19(100)
	No IPV	312 (69.8)	286 (100)	26 (18)	0 (0)	272 (56.3)	234 (91)	38 (18)	0 (0)
	Psychological IPV	93 (20.8)	0 (0)	93 (64)	0 (0)	172 (35.6)	24 (9)	148 (72)	0 (0)
	Physical and Psychological IPV	42 (9.4)	0 (0)	27 (18)	15 (100)	39 (8.1)	0 (0)	20 (10)	19 (100)
Protective Factors		Reference Group	Limited	Changing	Combined		Limited	Changing	Combined
Age *			29.8 (5.3)	27.4 (5.5)	27.5 (6.9)		30.1 (4.9)	29.3 (5.9)	29.4 (5.2)
Woman’s PTEs	None	1 or more	200 (47)	121 (58)	14 (74)		104 (29)	49 (16)	4 (15)
Family Composition	Couple	Single parent or multiple family	83 (20)	66 (32)	7 (37)		61 (17)	57 (19)	11 (41)
Number of Children	No child		151 (36)	65 (31)	5 (26)		120 (34)	77 (25)	7 (26)
	One		146 (35)	71 (34)	8 (42)		121 (34)	91 (30)	4 (15)
	Two		77 (18)	41 (20)	3 (16)		67 (19)	53 (18)	5 (19)
	Three or more		49 (12)	31 (15)	3 (16)		48 (13)	81 (27)	11 (41)
Can rely or confide in spouse	A lot or some	Little or not at all	25 (6)	40 (19)	12 (63)		28 (8)	54 (18)	14 (52)
Number of family can rely/confide in	Three or more	Two or fewer	74 (17)	38 (18)	11 (58)		155 (44)	171 (57)	16 (59)
Number of friends can rely/confide in	Three or more	Two or fewer	175 (41)	120 (58)	13 (68)		226 (63)	240 (79)	21 (78)
Partner PTEs	None		140 (42)	70 (40)	2 (11)		126 (41)	52 (21)	3 (12)
	One		96 (29)	43 (25)	6 (32)		66 (21)	63 (25)	8 (32)
	Two to three		70 (21)	46 (26)	7 (37)		78 (25)	80 (32)	9 (36)
	Four and more TEs		29 (9)	15 (9)	4 (21)		38 (12)	55 (22)	5 (20)
Financial Stress	None	1 or more	68 (16)	73 (35)	11 (58)		98 (28)	146 (48)	14 (52)
Living Difficulties	Mean		NA	NA	NA		1.5 (2.2)	1.9 (2.6)	5 (3.2)

Notes.

* Indicates changes in statistic format to Mean (SD). All variables were collected at Time 1, except ‘Partner PTEs’. NA, not available (not collected for Australian born women).

**Table 2.** Imputed data and goodness-of-fit indices for latent growth classes for Australian-born women (*n* = 650) and women from refugee backgrounds (*n* = 685).

	Australian-Born (*n* = 650)			Entropy (SD)		Conflict-Affected Countries (*n* = 685)			
	AIC (SD)	BIC (SD)	SS-BIC (SD)			AIC (SD)	BIC (SD)	SS-BIC (SD)	Entropy (SD)
**1 class** linear	2927 (27)		2930 (27)		**1 class** linear	3760 (27)	3774 (27)	3765 (27)	
quadratic	2928 (27)	2946 (27)	2934 (27)		quadratic	3761 (27)	3779 (27)	3767 (27)	
**2 class** linear	2632 (27)	2659 (27)	2640 (27)	0.751	**2 class** linear	3335 (28)	3362 (28)	3343 (28)	0.739
quadratic	2635 (27)	2671 (27)	2646 (27)	0.752	quadratic	3331 (28)	3367 (28)	3341 (28)	0.747
**3 class** linear	2578 (29)	2618 (29)	2589 (29)	0.76	**3 class** linear	3254 (25)	3294 (25)	3265 (24)	0.802
quadratic	2582 (29)	2636 (29)	2598 (29)	0.76	quadratic	3254 (25)	3308 (25)	3270 (25)	0.788
**4 class** linear	2569 (29)	2623 (29)	2584 (29)	0.801	**4 class** linear	3247 (26)	3302 (26)	3264 (26)	0.768
quadratic	2570 (29)	2642 (29)	2591 (29)	0.831	quadratic	3251 (26)	3323 (26)	3272 (26)	0.799
**5 class** linear	2568 (29)	2636 (29)	2588 (29)	0.801	**5 class** linear	3239 (27)	3307 (27)	3260 (27)	0.789
quadratic	2569 (28)	2659 (28)	2595 (28)	0.876	quadratic	3246 (27)	3337 (27)	3273 (27)	0.841
**6 class** linear	2566 (27)	2647 (28)	2590 (28)	831	**6 class** linear	3238 (28)	3320 (28)	3263 (28)	0.811
quadratic	2568 (28)	2675 (28)	2599 (28)	0.886	quadratic	3247 (27)	3326 (27)	3279 (27)	0.839

Notes. Linear and quadratic iterations of the model were performed with increasing numbers of latent classes and their model fit was compared to identify the best fitting model. Selecting the best model is based on both theoretical considerations and relative statistical fits between models. AIC = Akaike information criterion; BIC = Bayesian information criterion; SS-BIC = sample-size-adjusted Bayesian information criterion; VLMBLRT and BLRT not available for imputations. Number of imputations completed = 148/150. The three-class model showed the best model fit and/or most parsimonious classes.

**Table 3.** Three IPV latent classes based on posterior probabilities for observed and imputed data.

Latent Class			Australian-Born (*n* = 650)					Refugee Background (*n* = 685)		
Observed Data	*n* (%)	Intercept	*p*	Slope	*p*	*n* (%)	Intercept	*p*	Slope	*p*
Limited IPV	406 (63)	−3.05	<0.001	0.049	0.859	327 (48)	−5.797	<0.001	−0.086	0.793
Changing IPV	218 (33)	-	-	0.22	0.147	327 (48)	−2.373	0.005	0.001	0.996
Combined IPV	26 (4)	2.92	<0.001	1.75	0.053	32 (5)	-	-	2.348	0.196
**Imputed Data**										
Limited IPV	417 (64)	-	-	0.08	0.213	330 (48)	−3.423	0.128	0.043	0.177
Changing IPV	204 (31)	2.94	0.183	0.215	0.237	315 (46)	-	-	0.008	0.184
Combined IPV	29 (4)	5.44	0.177	2.23	0.56	41 (6)	1.526	0.205	6.511	0.416

Note. Shows the number of people and percentage of cohort in each latent class for both observed data and imputed data. The intercept shows the mean intercept with zero for the class. The coefficients for each class are (1) Limited IPV; (2) Changing IPV; and (3) Combined IPV. The slope shows the mean rate of change in IPV score across the three time points for that class. In each analysis, the model selects one class as a reference group, so the parameters are not estimated. The symbol (−) in table indicate that values for respective parameter is not estimated. Posterior probabilities refer to the probability that a given case falls into a given latent class.

**Table 4.** Adjusted odds ratios for protective factors associated with latent class membership for imputed data.

	Protective Factor		Refugee Background								
			Changing IPV			Combined IPV			Combined IPV		
			(Limited Reference Group)			(Limited IPV Reference Group)			(Changing IPV Reference Group)		
		Reference Group	AOR	95% CI	*p*	AOR	95% CI	*p*	AOR	95% CI	*p*
Age	older	younger	0.92	(0.88–0.96)	0.001	0.89	(0.82–0.97)	0.021	0.97	(0.89–1.05)	0.54
Woman’s PTEs (T1)	none	1+	0.73	(0.44–1.21)	0.231	0.56	(0.14–2.28)	0.359	0.77	(0.18–3.25)	0.729
Family composition (T1)	couple	single parent/multi-family	1.21	(0.72–2.03)	0.58	0.46	(0.19–1.13)	0.032	0.38	(0.16–0.94)	0.003
Number of children (T1)	2 or fewer	3+	0.29	(0.17–0.52)	<0.001	0.22	(0.08–0.62)	<0.001	0.74	(0.27–2.03)	0.567
Can rely/confide in partner (T1)	some–a lot	a little–not at all	0.37	(0.19–0.74)	<0.001	0.12	(0.05–0.34)	<0.001	0.33	(0.13–0.87)	0.001
No. family can rely/confide in (T1)	3 or more	2 or fewer	0.73	(0.49–1.08)	0.121	1.12	(0.47–2.64)	0.843	1.53	(0.64–3.66)	0.516
No. friends can rely/confide in (T1)	3 or more	2 or fewer	0.53	(0.36–0.83)	0.001	0.65	(0.24–1.75)	0.373	1.23	(0.45–0.34)	0.759
Partner’s PTEs (T2)	fewer	greater	0.62	(0.49–0.77)	<0.001	0.61	(0.38–0.98)	0.027	0.99	(0.61–1.60)	0.962
Financial stress (T1)	none	1+	0.67	(0.43–1.023)	0.055	0.62	(0.25–1.57)	0.282	0.93	(0.37–2.4)	0.902
Living difficulties (T1)	fewer	greater	0.99	(0.9–1.089)	0.833	0.72	(0.62–0.83)	<0.001	0.73	(0.63–0.85)	<0.001
	Protective Factor		Australian-Born								
			Changing IPV			Combined IPV			Combined IPV		
			(Limited IPV Reference Group)			(Limited IPV Reference Group)			(Changing IPV Reference Group)		
		Reference Group	AOR	95% CI	*p*	AOR	95% CI	*p*	AOR	95% CI	*p*
Age	older	younger	0.87	(0.86–0.91)	0.002	0.84	(0.82–0.94)	0.373	0.92	(0.89–1.03)	0.668
Woman’s PTEs (T1)	none	1+	0.36	(0.33–0.6)	0.029	0.22	(0.17–0.86)	0.851	0.34	(0.26–1.44)	0.726
Family composition (T1)	couple	single parent/multi-family	0.48	(0.42–0.88)	0.717	0.26	(0.2–1.04)	0.968	0.27	(0.21–1.18)	0.866
Number of children (T1)	2 or fewer	3+	0.34	(0.29–0.72)	0.41	0.18	(0.14–0.82)	0.806	0.24	(0.18–1.13)	0.901
Can rely/confide in partner (T1)	some–a lot	a little–not at all	0.11	(0.09–0.3)	<0.001	0.01	(0.01–0.03)	<0.001	0.01	(0.01–0.11)	<0.001
No. family can rely/confide in (T1)	some–a lot	a little–not at all	0.68	(0.6–1.31)	0.551	0.13	(0.1–0.55)	0.343	0.1	(0.01–0.42)	0.112
No. friends can rely/confide in (T1)	3 or more	2 or fewer	0.24	(0.22–0.42)	<0.001	0.14	(0.11–0.49)	0.159	0.33	(0.26–1.18)	0.847
Partner’s PTEs(T2)	fewer	greater	0.67	(0.63–0.88)	0.402	0.17	(0.15–0.41)	0.007	0.2	(0.17–0.46)	0.031
Financial stress(T1)	none	1+	0.22	(0.19–0.41)	<0.001	0.06	(0.05–0.22)	<0.001	0.15	(0.11–0.53)	0.266

Note: Continuous (age), count (living difficulties), and ordinal (partner trauma events) are shown as having more or less of the variable, while all other variables are dichotomous and are shown as protective factor and reference group.

## Data Availability

The data presented in this study are available on request from the corresponding author due to the sensitivity of the data contents. Requests to access the datasets should be directed to s.j.rees@unsw.edu.au.
