# Supplementary material for: Longitudinal Protective Factors against Intimate Partner Violence for Women Born in Australia and Women from Refugee Backgrounds
Source: Women (Basel). Author manuscript; Available in PMC 2026 Mar 11. (PMC12975033; doi:10.3390/women4030024)
Supplement: Supplementary Material [file NIHMS2092034-supplement-Supplementary_Material.pdf]

Multiple Imputation Procedure

Multiple Imputation Chain Equations (MICE) were used to impute missing data for the IPV variable. 100 imputations were performed each with 100 iterations. Variables related to patterns of missingness were used to improve imputation accuracy. These included: Education (1 = bachelor or more; 0 = diploma or less); housing status (0 = owner/renter; 1 = boarder/other); marital status (1= married/de facto; 0 = other); and the 12-item version of the World Health Organisation Disability Assessment Schedule 2.0 was used to measure disability related to a health condition (Andrews, Kemp, Sunderland, Von Korff, & Ustun, 2009) [60]. The WHODAS measures disability across six domains: Understanding and communication; self-care; mobility; interpersonal relationships; work and household roles; community and civic roles. Items are rated on a 5-point scale for level of difficulty from none (1) to extreme (5) with sum scores ranging from 0–48.

Below is a list of living difficulties that migrants and refugees who have arrived in Australia sometimes experience. During the past 12 months have any of the difficulties listed below been a problem for you in Australia?

NO PROBLEM AT ALL
A PROBLEM
A VERY SERIOUS PROBLEM

11.16 Communication difficulties
11.17 Discrimination
11.18 Separation from family 0
11.19 Worries about family back at home
11.20 Unable to return home in Emergency
11.21 No permission to work
11.22 Not being able to find work
11.23 Bad job conditions
11.24 Being in detention
11.25 Poor access to counselling services
11.26 Little Government help with welfare
11.27 Little help with welfare from Charities
11.28 Poverty
11.29 Isolation
11.30 Isolation that is intentionally caused by your husband/partner
11.31 Worry about being sent back to your country by the government
11.32 Worry about being sent back to your country by your husband
11.33 Worry that your husband might take your child or baby away from you
11.34 Worry that your husband might marry or live with another woman
11.35 Poor access to foods you like
11.36 Ever been a problem with Maher, dowry or bride price?

Table S1. Number of participants interviewed and missed out with retention rate for Australian born and women from refugee backgrounds across three time points.

Survey Time points	Australian born		Refugee backgrounds		All women	
	Participants interviewed n (% Retention)	Participants missed out n (% missed out)	Participants interviewed n (% Retention)	Participants missed out n (% missed out)	Participants interviewed n (% Retention)	Participants missed out n (% missed out)
**Time 1**	**650 (100)**	**0**	**685 (100)**	**0**	**1335 (100)**	**0**
Time 2	528 (81.2)	122 (18.8)	583 (85.1)	102 (14.9)	1111 (83.2)	224 (16.8)
Time 3	447 (68.8)	203 (31.2)	483 (70.5)	202 (29.5)	930 (69.7)	405 (30.3)
All three time points	***435 (66.9)***	***215 (33.1)***	***470 (68.6)***	***215 (31.4)***	***905 (67.8)***	***430 (32.2)***

Table S2. List of Potential Traumatic Events (PTEs).

(1) Kidnapping;
(2) Life-threatening automobile accident;
(3) Other life-threatening accidents;
(4) Life-threatening illness;
(5) Physical child abuse;
(6) Threatened with a weapon;
(7) Unexpected death of loved one;
(8) Child with life-threatening illness or injury;
(9) Traumatic event of loved one;
(10) Accidental serious injury or death of another person;
(11) Intentionally seriously injure, torture, or kill another person;
(12) Other extremely traumatic or life-threatening events;
(13) Traumatic event that you didn't report because you didn't want to talk about it;
(14) Unarmed civilian in conflict situation;
(15) Major natural disaster;
(16) Man-made disaster;
(17) Witness injury or death;
(18) Witness atrocities.

Table S3. Observed data Goodness-of-fit Indices for Latent Growth Classes for Women from Refugee Backgrounds and Australian born.

**Australian Born**						
	**AIC**	**BIC**	**SS-BIC**	**Entropy**	**VLMBLRT *p*-value**	**BLRT *p*-value**
**1 class** linear	**2394**	**2408**	**2398**			
quadratic	**2396**	**2414**	**2401**			
**2 class** linear	**2181**	**2207**	**2188**	**0.657**	**<0.001**	**<0.001**
quadratic	**2183**	**2219**	**2194**	**0.657**		
**3 class** linear	**2146**	**2186**	**2158**	**0.699**	**0.002**	**<0.001**
quadratic	**2149**	**2203**	**2165**	**0.700**		
**4 class** linear	**2141**	**2195**	**2157**	**0.729**		
quadratic	*					
**5 class** linear	**2141**	**2208**	**2160**	**0.770**		
quadratic	*					

	AIC	BIC	SS-BIC	Entropy
**6 class linear**	**2143**	**2224**	**2166**	**0.790**
quadratic	*			
**Refugee backgrounds**				
	**AIC**	**BIC**	**SS-BIC**	**Entropy**
**1 class linear**	**3085**	**3098**	**3089**	
quadratic	**3087**	**3105**	**3092**	
**2 class linear**	**2772**	**2799**	**2780**	**0.678**
quadratic	**2773**	**2809**	**2784**	**0.680**
**3 class linear**	**2730**	**2770**	**2742**	**0.748**
quadratic	**2734**	**2788**	**2750**	**0.749**
**4 class linear**	**2772**	**2777**	**2739**	**0.736**
quadratic	*			
**5 class linear**	**2711**	**2779**	**2731**	**0.775**
quadratic	*			
**6 class linear**	**2710**	**2792**	**2735**	**0.800**
quadratic	*			

Notes. AIC = Akaike information criterion; BIC = Bayesian information criterion; SS-BIC = sample size-adjusted Bayesian information criterion; * = convergence issues.

Notebook for multiple imputation procedures and Mplus syntax for LCGA

```
(watch <- australian_born %>%
 mutate(group = 0) %>%
 full_join(migrant_only %>%
           mutate(group = 1)) %>%
  mutate_all(~case_when(.==999~NA_real_,TRUE~.)) %>%
  mutate_at(vars("STUDYID","group"),~as.factor(.)) %>%
  mutate(across(where(is.numeric),
         ~ case_when(is.na(.)==TRUE~0,TRUE~1))) %>%
  relocate(STUDYID,group) %>%
  group_by(group))
```

Joining, by = c("STUDYID", "T1COB", "T1Age", "T1Marital", "T1Refugee", "T1Housing_dichot", "T1Fin_2", "T1incom", "T1HMLES", "T1educ_dichot", "T2EngProf", "T1NOCHild", "T1_FAMCOMP_Dichot", "T1SpsRelCon_dichot", "T1FamFrndCont", "T1famrelycon", "T1frnrelycon", "T1FrndFamRely", "T1FrndFamConfy", "T2SocIsoltn", "T1TEall", "T1TEcat", "T1WHODAS", "T2WHODAS", "T2LivDifCltT", "T1Q1_27ADAPT", "T1ADAPT", "T1WMRights", "T1MnHitWf", "T2WMRights", "T2MnHitWf", "HusbTEcat_T2", "T2Employ_Husb", "IPV_Trajct", "IPV_Traj_Group", "T1_IPV", "T2_IPV", "T3_IPV", "T1Q1_9AGE", "group")

STUDYID <fctr>	group <fctr>	T1COB <dbl>	T1Age <dbl>	T1Marital <dbl>	T1Refugee <dbl>	T1Housing_dichot <dbl>	T1Fin_2 <dbl>	T1incom <dbl>	T1HMLES <dbl>
4	0	1	1	1	1	1	1	1	1

STUDYID <fctr>	group <fctr>	T1COB <dbl>	T1Age <dbl>	T1Marital <dbl>	T1Refugee <dbl>	T1Housing_dichot <dbl>	T1Fin_2 <dbl>	T1incom <dbl>	T1HMLES <dbl>
5	0	1	1	1	1	1	1	1	1
13	0	1	1	1	1	1	1	1	1
16	0	1	1	1	1	1	1	1	1
18	0	1	1	1	1	1	1	1	1
19	0	1	1	1	1	1	1	1	1
20	0	1	1	1	1	1	1	1	1
22	0	1	1	1	1	1	1	1	1
24	0	1	1	1	1	1	1	1	1
81	0	1	1	1	1	1	1	1	1

Next

123456

...

134

Previous

1-10 of 1,335 rows | 1-10 of 40 columns

what is the total number of complete cases?

Hide

```
watch %>%
  select(STUDYID,T1_IPV,T2_IPV,T3_IPV) %>%
  ungroup() %>% group_by(STUDYID,group) %>%
  mutate(complete = case_when(sum(T1_IPV,T2_IPV,T3_IPV)==3~1,TRUE~0)) %>%
  ungroup() %>% group_by(group) %>%
  count(complete) %>%
  mutate(`%` = round((n*100/sum(n)),1))
```

Adding missing grouping variables: `group`

group <fctr>	complete <dbl>	n <int>	% <dbl>
0	0	215	33.1
0	1	435	66.9
1	0	215	31.4

group	complete	n	%
<fctr>	<dbl>	<int>	<dbl>
1	1	470	68.6

4 rows

sum outcome vars and calculate percentage missingness by group by wave

Hide

```
watch %>%
  select(T1_IPV,T2_IPV,T3_IPV) %>%
  summarise(across(where(is.numeric),sum,na.rm=FALSE)) %>%
  pivot_longer(cols=2:4,names_to="wave",values_to="count") %>%
  mutate(wave = case_when(str_detect(wave,"T1_IPV")~1,
                          str_detect(wave,"T2_IPV")~2,
                          TRUE~3),
         `%` = case_when(group==0~round((count/650*100),1),
                         group==1~round((count/685*100),1)))
```

```
Adding missing grouping variables: `group`
```

group	wave	count	%
<fctr>	<dbl>	<dbl>	<dbl>
0	1	650	100.0
0	2	528	81.2
0	3	447	68.8
1	1	685	100.0
1	2	583	85.1
1	3	483	70.5

6 rows

calculate missingness for auxiliary vars

Hide

```
watch %>%
  select(names(watch)[3:36]) %>%
  summarise(across(where(is.numeric),sum,na.rm=FALSE)) %>%
  pivot_longer(cols=2:35,names_to="vars",values_to="count") %>%
  mutate(`%` = case_when(group==0~round((count/650*100),1),
                         group==1~round((count/685*100),1))) %>%
```

```
  arrange(desc(`%`))
```

Adding missing grouping variables: `group`

group	vars	count	%
<fctr>	<chr>	<dbl>	<dbl>
0	T1COB	650	100.0
0	T1Age	650	100.0
0	T1Marital	650	100.0
0	T1Refugee	650	100.0
0	T1Housing_dichot	650	100.0
0	T1Fin_2	650	100.0
0	T1incom	650	100.0
0	T1educ_dichot	650	100.0
0	T1NOCHild	650	100.0
0	T1_FAMCOMP_Dichot	650	100.0

Next

1234567

Previous

1-10 of 68 rows

prepare gender attitudes data for inclusion in multiple imputation

Hide

```
gender_attitudes <- read_spss("datafiles/revised_11-10-21/gender-attitudes_raw.sav",
                              .name_repair ="universal") %>%
  sjlabelled::remove_all_labels() %>%
  janitor::clean_names() %>%
  set_names(names(.) %>% str_replace_all(.,"^x[:digit:]","") %>%
            str_replace_all("^[:punct:]","") %>%
            str_replace_all("^[:digit:][:punct:]","")) %>%
  rename("STUDYID" = studyid)
```

New names:

* `@1obey` -> ..1obey

* `@2withinfamily` -> ..2withinfamily

* `@3showboss` -> ..3showboss

```
* `@4choose_friendsR` -> ..4choose_friendsR

* `@5_obl_sex` -> ..5_obl_sex

* ...
```

Hide

```
domain_A <- c("obey","withinfamily","showboss","choose_friends_r","obl_sex","others_intervene_r")
domain_B <- c("hit_housework","hit_disobey","hit_refuse_sex","hit_ask_girlfriends","hit_suspect",
              "hit_unfaithful")
domain_C <- c("refuse_want","refuse_drunk","refuse_sick","refuse_mistreat")


(gender_attitudes %<>%
  rowwise() %>%
  # make domain vars and recode C
  mutate(domain_A = sum(c_across(domain_A)),
         domain_B = sum(c_across(domain_B)),
         domain_C = sum(c_across(domain_C))) %>%
          # recode(.,`0`=4,`1`=3,`3`=1,`4`=0)) %>% # redundant
  # dichotomise domain scores
  mutate(domain_A = case_when(domain_A<3~0,TRUE~1),
         domain_B = case_when(domain_B<1~0,TRUE~1),
         domain_C = case_when(domain_C<1~0,TRUE~1)) %>%
  # make attitudes composite
  mutate(composite = sum(domain_A,domain_B,domain_C)) %>%
  arrange(STUDYID) %>%
  mutate_all(~as.factor(.)) %>%
  select(STUDYID,domain_A:composite))
```

```
Note: Using an external vector in selections is ambiguous.
ℹ Use `all_of(domain_A)` instead of `domain_A` to silence this message.
ℹ See <https://tidyselect.r-lib.org/reference/faq-external-vector.html>.
This message is displayed once per session.
Note: Using an external vector in selections is ambiguous.
ℹ Use `all_of(domain_B)` instead of `domain_B` to silence this message.
ℹ See <https://tidyselect.r-lib.org/reference/faq-external-vector.html>.
```

This message is displayed once per session.

Note: Using an external vector in selections is ambiguous.

i Use `all_of(domain_C)` instead of `domain_C` to silence this message.

i See <https://tidyselect.r-lib.org/reference/faq-external-vector.html>.

This message is displayed once per session.

**STUDYID**<br><fctr>	**domain_A**<br><fctr>	**domain_B**<br><fctr>	**domain_C**<br><fctr>	**composite**<br><fctr>
1	0	1	0	1
4	0	0	0	0
5	0	0	0	0
7	0	0	1	1
11	0	0	0	0
12	1	0	0	1
13	0	0	0	0
14	0	0	0	0
16	0	0	0	0
18	0	0	0	0

Next

123456

...

103

Previous

1-10 of 1,024 rows

Hide

```
migrant_attitudes <- read_spss("datafiles/revised_11-10-21/gender-attitudes_migrants.sav",
                               .name_repair ="universal") %>%
  sjlabelled::remove_all_labels() %>%
  janitor::clean_names() %>%
  set_names(names(.) %>% str_replace_all(.,"^x[:digit:]","") %>%
            str_replace_all("^[:punct:]","") %>%
            str_replace_all("^[:digit:][:punct:]","")) %>%
  rename("STUDYID" = studyid)
```

```
New names:
* `@1obey` -> ..1obey
* `@2withinfamily` -> ..2withinfamily
* `@3showboss` -> ..3showboss
* `@4choose_friendsR` -> ..4choose_friendsR
* `@5_obl_sex` -> ..5_obl_sex
* ...
```

Hide

```
(migrant_attitudes %<>%
  rowwise() %>%
  # make domain vars and recode C
  mutate(domain_A = sum(c_across(domain_A)),
         domain_B = sum(c_across(domain_B)),
         domain_C = sum(c_across(domain_C))) %>%
          # recode(.,`0`=4,`1`=3,`3`=1,`4`=0)) %>% # redundant
  # dichotomise domain scores
  mutate(domain_A = case_when(domain_A<3~0,TRUE~1),
         domain_B = case_when(domain_B<1~0,TRUE~1),
         domain_C = case_when(domain_C<1~0,TRUE~1)) %>%
  # make attitudes composite
  mutate(composite = sum(domain_A,domain_B,domain_C)) %>%
  arrange(STUDYID) %>%
  mutate_all(~as.factor(.)) %>%
  select(STUDYID,domain_A:composite))
```

STUDYID <fctr>	domain_A <fctr>	domain_B <fctr>	domain_C <fctr>	composite <fctr>
1	0	1	0	1
7	0	0	1	1
11	0	0	0	0
12	1	0	0	1
14	0	0	0	0
21	0	0	0	0

STUDYID <fctr>	domain_A <fctr>	domain_B <fctr>	domain_C <fctr>	composite <fctr>
26	0	0	0	0
28	1	0	0	1
29	1	0	0	1
31	0	1	0	1

Next

123456

...

69

Previous

1-10 of 685 rows

check composites are the same across datasets

Hide

```
gender_attitudes %>%
  full_join(migrant_attitudes,
       by = c("STUDYID","domain_A","domain_B","domain_C")) %>%
  mutate(test = composite.x==composite.y) %>%
  filter(test==FALSE)
```

0 rows

anti_join migrants into raw to get Australian-born database

Hide

```
(australian_attitudes <- gender_attitudes %>%
  anti_join(migrant_attitudes,
       by = c("STUDYID")))
```

STUDYID <fctr>	domain_A <fctr>	domain_B <fctr>	domain_C <fctr>	composite <fctr>
4	0	0	0	0
5	0	0	0	0
13	0	0	0	0
16	0	0	0	0

STUDYID <fctr>	domain_A <fctr>	domain_B <fctr>	domain_C <fctr>	composite <fctr>
18	0	0	0	0
19	0	0	0	0
20	1	0	0	1
22	0	0	0	0
24	1	0	0	1
81	0	0	0	0

Next

123456

...

45

Previous

1-10 of 446 rows

check for any duplicate STUDYID's

Hide

```
gender_attitudes %>%
  filter(duplicated(STUDYID) | duplicated(STUDYID,fromLast=TRUE))
```

0 rows

mutiple imputation

make custom function to identify columns containing NA values for multiple imputation

Hide

```
# function to identify incomplete cases column-wise
ics <- function (df) {
  colnames(df)[colSums(is.na(df)) > 0]
}
```

prepare dataframes for MICE procedure, run MICE and export estimates

Hide

```
# AUSTRALIAN BORN
ab_imp <- australian_born %>%
```

```
  select(STUDYID,T1WHODAS,T1Housing_dichot,T1educ_dichot,T1Marital,T1_IPV,T2_IPV,
      T3_IPV,T1Q1_9AGE,T1_FAMCOMP_Dichot,T1SpsRelCon_dichot,T1NOCHild,T1Fin_2,
      T1famrelycon,T1frnrelycon,T1TEcat,HusbTEcat_T2) %>%
  mutate_all(as.factor) %>%
  mutate(T1WHODAS=as.integer(T1WHODAS)) %>%
  rename(T1Housing=T1Housing_dichot,T1Education=T1educ_dichot,T1Age=T1Q1_9AGE,
      T1FamilyComp=T1_FAMCOMP_Dichot,T1NoChild=T1NOCHild,T1FamilyRC=T1famrelycon,
      T1FriendRC=T1frnrelycon,T2SpouseTEs=HusbTEcat_T2,T1SpouseRC=T1SpsRelCon_dichot,
      T1FinancialStress=T1Fin_2,T1TEs=T1TEcat) %>%
  mutate_at(vars(T1_IPV,T2_IPV,T3_IPV),
       ~ str_replace_all(.,"999",NA_character_) %>%
         as.factor(.)) %>%
  full_join(australian_attitudes,by = "STUDYID")

# which variables require imputation?
# T2_IPV,T3_IPV,T2SpouseTEs,T1SpousePhysical,T2SpousePhysical
ics(ab_imp)

# we run the mice code with 0 iterations
ab_imp_0 <- mice(ab_imp, maxit=0)

# create predictor matrix
ab_predM = ab_imp_0$predictorMatrix
ab_predM[, c("STUDYID","T1WHODAS","T1Marital","T1Housing","T1Education","T1Age",
          "T1TEs","T1FamilyComp","T1NoChild","T1FamilyRC","T1FriendRC","T1SpouseRC",
          "T1FinancialStress","domain_A","domain_B","domain_C","composite")] <- 0
ab_meth = ab_imp_0$method

# specify a separate imputation model for variables of interest
ab_poly <- c("T1_IPV","T2_IPV","T3_IPV","T2SpouseTEs") # ordered categorical

# turn methods matrix into the specified imputation models
ab_meth[ab_poly] = "polr"
```

```
ab_imp100_100 <- mice(ab_imp,
                   m=100,
                   maxit = 100,
                   predictorMatrix = ab_predM,
                   method = ab_meth,
                   print = FALSE)
```

```
New names:
* STUDYID -> STUDYID...1
* T1WHODAS -> T1WHODAS...2
* T1Housing -> T1Housing...3
* T1Education -> T1Education...4
* T1Marital -> T1Marital...5
* ...
```

STUDYID.0 <fctr>	T1WHODAS.0 <int>	T1Housing.0 <fctr>	T1Education.0 <fctr>	T1Marital.0 <fctr>	T1_IPV.0 <fctr>	T2_IPV.0 <fctr>	T3_IPV.0 <fctr>	T1Age.0 <fctr>
4	11	0	0	1	2	2	2	25
5	7	0	0	1	2	1	2	20
13	5	0	1	1	1	1	2	30
16	17	0	0	1	1	1	2	35
18	15	0	1	1	2	1	1	32
19	5	0	0	1	1	*NA*	1	41
20	6	0	0	1	2	*NA*	*NA*	20
22	5	0	0	1	3	2	2	43
24	5	0	0	2	1	1	1	28
81	28	0	1	1	2	1	*NA*	25

Next

123456

...

65

Previous

1-10 of 650 rows | 1-9 of 1717 columns

re-run MICE for migrant only data

Hide

```
# MIGRANT ONLY
mo_imp <- migrant_only %>%
  select(STUDYID,T1WHODAS,T1Housing_dichot,T1educ_dichot,T1Marital,T1_IPV,T2_IPV,
         T3_IPV,T2LivDifCltT,T1Q1_9AGE,T1_FAMCOMP_Dichot,T1SpsRelCon_dichot,
         T1NOCHild,T1Fin_2,T1famrelycon,T1frnrelycon,T1TEcat,HusbTEcat_T2) %>%
  mutate_all(as.factor) %>%
  mutate(T1WHODAS = as.integer(T1WHODAS)) %>%
  rename(T1Housing=T1Housing_dichot,T1Education=T1educ_dichot,T1Age=T1Q1_9AGE,
         T1FamilyComp=T1_FAMCOMP_Dichot,T2LivingDiff=T2LivDifCltT,T1NoChild=T1NOCHild,
         T1FamilyRC=T1famrelycon,T1FriendRC=T1frnrelycon,T2SpouseTEs=HusbTEcat_T2,
         T1SpouseRC=T1SpsRelCon_dichot,T1FinancialStress=T1Fin_2,T1TEs=T1TEcat) %>%
  mutate_at(vars(T1_IPV,T2_IPV,T3_IPV),~str_replace_all(.,"999",NA_character_) %>% as.factor(.)) %>%
  mutate_at(vars(T2LivingDiff),~as.numeric(.)) %>%
  full_join(migrant_attitudes,by = "STUDYID")

# which variables require imputation?
ics(mo_imp)
# T2_IPV,T3_IPV,T2LivingDiff,T2SpouseTEs

# we run the mice code with 0 iterations
mo_imp_0 <- mice(mo_imp, maxit=0)

# create predictor matrix
mo_predM = mo_imp_0$predictorMatrix
mo_predM[, c("STUDYID","T1WHODAS","T1Marital","T1Housing","T1Education","T1Age",
           "T1TEs","T1FamilyComp","T1NoChild","T1FamilyRC","T1FriendRC","T1SpouseRC",
           "T1FinancialStress","domain_A","domain_B","domain_C","composite")] <- 0
mo_meth = mo_imp_0$method

# specify a separate imputation model for variables of interest
mo_poly <- c("T1_IPV","T2_IPV","T3_IPV","T2SpouseTEs") # ordered categorical
mo_cont <- c("T2LivingDiff") #continuous
```

```
# turn methods matrix into the specified imputation models
mo_meth[mo_poly] = "polr"
mo_meth[mo_cont] = "midastouch"


mo_imp100_100 <- mice(mo_imp,
                m=100,
                maxit = 100,
                predictorMatrix = mo_predM,
                method = mo_meth,
                print = FALSE)
```

```
New names:
* STUDYID -> STUDYID...1
* T1WHODAS -> T1WHODAS...2
* T1Housing -> T1Housing...3
* T1Education -> T1Education...4
* T1Marital -> T1Marital...5
* ...
```

STUDYID.0 <fctr>	T1WHODAS.0 <int>	T1Housing.0 <fctr>	T1Education.0 <fctr>	T1Marital.0 <fctr>	T1_IPV.0 <fctr>	T2_IPV.0 <fctr>	T3_IPV.0 <fctr>	STUDYID.1 <fctr>
1	9	0	0	2	2	*NA*	*NA*	1
7	5	0	0	1	1	1	*NA*	7
11	3	0	1	2	2	2	2	11
12	14	0	1	1	1	2	2	12
14	3	0	0	1	2	1	1	14
21	2	0	1	1	1	1	1	21
26	4	0	0	2	3	2	*NA*	26
28	22	0	1	1	1	1	1	28
29	6	0	1	1	1	1	1	29
31	5	0	1	1	1	2	2	31

Next

123456

...

69

Previous

1-10 of 685 rows | 1-9 of 808 columns

MPLUS syntax

Hide

```
DATA: FILE IS data.dat;
TYPE = IMPUTATION;
VARIABLE: NAMES ARE
STUDYID
T1WHODAS
T1Housing
T1Education
T1Marital
T1_IPV
T2_IPV
T3_IPV
T1Age
T1FamilyComp
T1SpouseRC
T1NoChild
T1FinancialStress
T1FamilyRC
T1FriendRC
T1TEs
T2SpouseTEs
T1SpPhysical
T2SpousePhysical ;

 USEVARIABLES = T1_IPV
 T2_IPV
 T3_IPV  T1Age R_Fin_Str  RCHILD_2 R_SP_RC RFAMCOMP
 RFAM_REL RFR_REL R_WTE_2 R_SP_TE;
 CLASSES = LCGA (3);
```

```
  categorical = T1_IPV
  T2_IPV
  T3_IPV;

auxiliary =   T1Age (R3STEP);
auxiliary =   R_WTE_2 (R3STEP);
auxiliary =   RFAMCOMP (R3STEP);
auxiliary =   RCHILD_2 (R3STEP);
auxiliary =   R_SP_RC (R3STEP);
auxiliary =   RFAM_REL (R3STEP);
auxiliary =   RFR_REL (R3STEP);
auxiliary =   R_SP_TE (R3STEP);
auxiliary =   R_Fin_Str (R3STEP);

    MISSING = ALL (999);

Define:

      R_Age = T1Age*(-1);

     IF (T1FinancialStress EQ 1) then  R_Fin_Str = 0;
       IF (T1FinancialStress EQ 0) then  R_Fin_Str = 1;

       IF (T1FamilyComp EQ 1) then  RFAMCOMP= 0;
       IF (T1FamilyComp EQ 0) then  RFAMCOMP = 1;

       IF (T1NoChild LE 2) THEN RCHILD_2 = 1;
         IF (T1NoChild GE 3) THEN RCHILD_2 = 0;

        IF (T1TEs EQ 0) THEN R_WTE_2 = 1;
          IF (T1TEs GE 1) THEN R_WTE_2 = 0;

       IF (T1FamilyRC EQ 1) then  RFAM_REL= 0;
```

```
    IF (T1FamilyRC EQ 0) then  RFAM_REL = 1;


    IF (T1FriendRC EQ 1) then  RFR_REL= 0;
    IF (T1FriendRC EQ 0) then  RFR_REL = 1;


   R_SP_TE = T2SpouseTEs*(-1);


   R_SP_RC = T1SpouseRC*(-1);


ANALYSIS: TYPE = MIXTURE;


model:
    %overall%
    i s | T1_IPV@0   T2_IPV@1 T3_IPV@3;



OUTPUT: sampstat stdyx modindices TECH1 TECH8 TECH10 CINTERVAL;
```
